# The MOMANT study, a caregiver support programme with activities at home for people with dementia: results of a randomised controlled trial

**DOI:** 10.1186/s12877-026-07634-0

**Published:** 2026-05-20

**Authors:** Sanne C.E. Balvert, Elke Butterbrod, R. M. Dröes, Martijn W. Heymans, Erik J.A. Scherder, Maarten V. Milders

**Affiliations:** 1 Department of Clinical Neuropsychology, Faculty of Behavioural and Movement Sciences, Vrije Universiteit Amsterdam, Amsterdam, Netherlands; 2 Department of Neurosurgery, Elisabeth-Tweesteden Hospital, Tilburg, Netherlands; 3 Department of Psychiatry, Amsterdam University Medical Centres, location VU University Medical Centre, Amsterdam, Netherlands; 4 Amsterdam Public Health Research Institute, Amsterdam, Netherlands; 5 Department of Epidemiology and Data Science, Amsterdam University Medical Centres, location VU University Medical Centre, Amsterdam, Netherlands

**Keywords:** Dementia, Caregiver, Activities, Quality of life, Psychosocial intervention

## Abstract

**Background:**

Increasing numbers of persons with dementia are cared for at home by informal caregivers, underscoring the need for caregiver support. This study evaluated the MOMANT intervention: a psychosocial training programme for informal caregivers consisting of 6 group sessions led by healthcare professionals covering education on dementia, managing changes in behaviour and communication, and coping with caregiver burden. In addition, informal caregivers were trained to actively engage the person with dementia in stimulating activities at home. The primary aim was to improve the quality of life for both the informal caregiver and the person with dementia.

**Methods:**

This randomised controlled trial compared outcomes in 172 dyads (*n* = 74/43% intervention, *n* = 98/57% control continuing usual care). Training took place in small groups led by healthcare professionals at Dutch dementia daycare and Meeting Centres. Randomisation occurred at the centre level. Participants were assessed at baseline and at 3- and 6-month follow-up. Outcomes included [instrumental] activities of daily living ([I]ADL) dependency, global functioning, and activity engagement (person with dementia only); sense of competence, caregiving experiences, and mood (caregiver only); quality of life and healthcare usage (both). Caregivers and healthcare professionals also evaluated the intervention in surveys. Outcomes were analysed over time using longitudinal mixed models (intention-to-treat), with group by time interaction (control as reference), α = 0.05. Subsequently, effects between intervention and control groups were compared at 3 and 6 months.

**Results:**

Longitudinal mixed models showed no significant interactions for most outcomes, indicating that the trajectories of the outcomes of the intervention and control groups did not differ over time (*p*’s > .05). However, compared to the control group, persons with dementia in the intervention group engaged in more activities (t=-2.38, *p* = .02), and caregivers in the intervention group reported more positive caregiving experiences (t=-2.16, *p* = .03) at the 3-month follow-up. Subjective evaluations of the intervention were positive: caregivers considered the content useful and reported improved dyadic interactions.

**Conclusion:**

This study demonstrates the potential of a psychosocial caregiver-led intervention to increase activity engagement of the person with dementia and enhance positive caregiving experiences. Although most outcomes showed no significant changes, positive evaluations of the intervention highlighted the benefits of information, emotional support, and practical training for informal caregivers.

**Trial registration:**

The trial was registered at the Dutch Trial Register (NTR6643) August 22nd, 2017.

**Supplementary Information:**

The online version contains supplementary material available at 10.1186/s12877-026-07634-0.

## Introduction

Dementia is a growing global public health challenge, currently affecting approximately 50 million people worldwide, with this number projected to rise to 152 million by 2050 [1]. As most people with dementia live at home, much of the care is provided by informal caregivers, typically spouses or family members [2]. Informal caregivers play an essential role for the person with dementia, often attending to their medical needs, safety issues, and daily care (e.g. feeding, bathing, and changing), as well as providing emotional and social support [3]. However, providing this care is challenging and has been associated with high levels of stress, anxiety, and depression [4]. As dementia progresses, cognitive decline is accompanied by behavioural and psychological changes, which are also influenced by the person’s coping style and social environment, resulting in an increased need for support that places significant demands on informal caregivers [4]. According to the World Alzheimer Report (2024), 59% of family caregivers reported experiencing high or very high emotional stress, and 38% reported high physical stress. Dementia caregivers also showed higher rates of depression and anxiety compared to non-dementia caregivers [5]. In the Netherlands, 70% of people with dementia currently live at home, supported by informal caregivers who spend an average of 37 h per week on caregiving [2]. More than half (56%) of these caregivers reported feeling burdened, with 43% experiencing a fairly high burden, 10% a very high burden, and 3% feeling overburdened [2]. Additionally, 69% stated that they were the sole caregiver, often providing care day and night while struggling to balance their caregiving responsibilities with their own daily activities [2]. At the same time, the availability of professional healthcare is decreasing [6].

Given the increasing demand on informal caregivers, home-based psychosocial interventions to support caregivers, and consequently reduce caregiver burden are highly needed. Multi-component psychosocial interventions consist of various therapeutic strategies aimed at the informal caregiver, the person with dementia, or both to improve well-being and daily functioning [7]. Providing person-centred care has been shown to be associated with a higher quality of life for both persons with dementia and their informal caregivers, as well as a higher quality of care [8]. Interventions appear to be particularly effective when activities are tailored to the interests and abilities of the person with dementia, helping them to remain physically and socially engaged. Maintaining engagement fosters a sense of meaning and purpose, which can ultimately improve the quality of life of the person with dementia [9, 10]. As the disease progresses, caregivers dedicate an increasing amount of time to providing assistance and supervision, which can lead to heightened stress and burden [11]. If some of this time is spent engaging in pleasant and meaningful activities together, it may serve as a protective factor against caregiver burden and also help to reduce behavioural symptoms in the person with dementia [12, 13]. Additionally, shared activities can create opportunities for meaningful and intimate moments and reciprocity, strengthening the emotional connection between caregivers and persons with dementia, even in non-spousal relationships [14]. Peer support also appears to be particularly beneficial, as it can improve caregivers’ skill development, coping strategies, and self-management [3, 15, 16]. Peer support can significantly enhance caregivers’ well-being by reducing feelings of isolation. This is especially important because many caregivers lack social contacts and experience social isolation due to time constraints, limited opportunities to socialise, and the stigma surrounding dementia, which can lead to family and friends distancing themselves [17–19].

However, many of these interventions remain difficult to implement at a large scale because they tend to be labour-intensive and costly, and rely on highly trained professionals [20, 21]. There is some evidence that caregivers can be trained to deliver key intervention components themselves, particularly when combined with minimal professional guidance [21], but this remains underexplored. In recent years, there has been a growing trend to combine activity-based interventions with various psychoeducational programmes for the caregiver and these combined interventions have demonstrated positive effects on reducing caregiver burden, can be delivered in group settings to include peer support, and can lower the cost of intervention [22, 23]. Taken together, this suggests that caregiver-led, activity-based interventions, particularly as a multi-component intervention, offer a promising approach to provide flexible, cost-effective support in dementia care. Building on these insights, the present study explored whether a caregiver-led approach that integrated psycho-educational and activity-based elements could offer effective support for informal caregivers of community-dwelling people with dementia.

The current study evaluated the effect of the MOMANT intervention (in Dutch: “Mantelzorg Ondersteuning Met ActiviteiteN Thuis”, translated as: “Caregiver Support with Activities at Home”), a caregiver support programme aimed at promoting activities at home for people with dementia. The MOMANT intervention combined two evidence-based approaches to caregiver support: (i) multi-component interventions, which have been shown to improve caregiver well-being and delay the institutionalisation of the person with dementia, and (ii) activity-based interventions, in which caregivers were trained to engage the person with dementia in meaningful, stimulating activities, which have been shown to improve the quality of life for both [8, 10, 12, 21, 24, 25]. Multicomponent interventions combine two or more approaches (e.g. psychoeducation, skills training, and coping strategies) and have demonstrated effectiveness in reducing caregiver burden and improving caregiver well-being [22]. Such interventions may be based on various theoretical frameworks, including stress-coping models, cognitive-behavioural approaches, and person-centred perspectives that emphasize resilience and positive aspects of caregiving [22, 26]. Activity-based components, such as engaging the person with dementia in meaningful shared activities, are grounded in the principle that social and cognitive stimulation can positively affect the wellbeing of both the person with dementia and the informal caregiver [27, 28]. By training informal caregivers to engage the person with dementia in stimulating activities, the additional demands on healthcare professionals could be minimal. The MOMANT intervention aimed to improve caregivers’ well-being through education and peer support, and by encouraging enjoyable activities that the informal caregiver could engage in together with the person with dementia [29]. Previous research has indicated that this approach also has positive effects on the caregiver’s sense of competence and their caregiving experience [15, 25, 30, 31]. The main aim of this study was to investigate whether the intervention could improve the quality of life of the informal caregiver and the person with dementia, as well as caregivers’ feelings of competence, caregiving experience, and mood.

## Methods

### Study design and setting

The study design was a single blind cluster randomised controlled trial. Outcomes were compared between two groups; an intervention group who participated in the MOMANT intervention and a control group who continued with usual care. Participants were recruited throughout the Netherlands by healthcare professionals of daycare centres or Meeting Centres. Meeting Centres offer professional person-centred, psychosocial support for people with mild to moderately severe dementia, who can participate in recreational or creative activities under supervision of healthcare professionals, volunteers and their informal caregivers. Dementia daycare centres focus primarily on providing structured daytime activities and supervision for people with moderate to advanced dementia, thereby providing respite care for informal caregivers. Study sites were selected based on their target population (persons with dementia living at home and their informal caregivers), their geographical locations (to ensure an even distribution across the country), and the availability of staff.

Randomisation occurred at the level of the dementia daycare centre or Meeting Centre, using a computer-generated allocation sequence. All dyads recruited at a centre were enrolled in the condition to which that centre had been allocated. Data were collected at baseline, prior to the start of the intervention, and at 3 and 6-month follow-up.

The MOMANT project (registered at the Dutch Trial Register; Trial ID, NTR6643) was coordinated at the Vrije Universiteit Amsterdam and involved organisations for the care and welfare of the elderly across the Netherlands. The project took place between September 2017 and June 2022. The study was conducted and is reported in accordance with the CONSORT (Consolidated Standards of Reporting Trials) guidelines. A completed CONSORT checklist is provided as an additional file (Supplementary File 1). Further details of the MOMANT study, including on participant recruitment, can be found elsewhere [29].

### Participants

Study participants were dyads consisting of an informal caregiver and the person with dementia for whom they cared.

Informal caregivers were spouses, relatives or friends who cared for and supported the person with dementia without receiving payment. If the dyad did not live together, the informal caregiver had to visit the person with dementia at least three times a week to be eligible for participation. The person with dementia was living at home, and had to have a formal clinical diagnosis of dementia or experience cognitive impairments affecting their daily functioning such that dementia was strongly suspected. Type of dementia was not an inclusion criterion.

Exclusion criteria for both the caregiver and the person with dementia were major mental or physical illness, such as major depression, stroke or Korsakov, that would affect their ability to participate in the intervention or complete the assessments. The person with dementia should not have started with dementia-specific medication less than 6 months before inclusion. Participation in another intervention study during the time that the dyad participated in the current study was a further exclusion criterion.

### Ethical approval

This study was conducted according to the principles of the Declaration of Helsinki. All participants gave written informed consent prior to participation. For persons with dementia, capacity to provide informed consent was assessed by the research team together with the informal caregiver. Only individuals who were deemed capable of understanding the study information and providing informed consent were included in the study. The Medical Ethics committee of the Amsterdam University Medical Centres, location Vrije Universiteit Medical Centre, considered this study as not subjected to the Medical Research Involving Human Subjects Act and the Ethics Committee of the Faculty of Behavioural and Movements Sciences of the Vrije Universiteit Amsterdam approved the study (VCWE-2017-015).

### Procedure

The aim and procedure of the research was explained to all potential participants: (i) when they were first contacted by telephone by the research assistant, and (ii) when they received written information about the study from their dementia case manager or local healthcare professional. Potential participants then had two weeks to decide whether they were willing to participate in the study during which time they could contact the lead researcher for further information. If they decided to take part, a baseline measurement meeting was scheduled and both the person with dementia and the informal caregiver gave informed consent at the start of this meeting.

Data from participants in the intervention condition and control condition were collected on three occasions by trained researchers, who were blind to the condition to which the participants had been assigned. The data were collected at participants’ homes or if they preferred at the dementia daycare centre or Meeting Centre. During the home visits, outcome measures were administered by the research assistant in interview format. Outcome measures were preferably administered with both members of the dyad present. To facilitate honest answers from the informal caregiver, all questionnaires worked with answer sheets so that they could indicate their answer discreetly if preferred. The responses were subsequently entered into a database under a subject number; any personal information was removed and stored separately.

Part of the study period overlapped with the COVID-19 pandemic. During lockdown periods, all intervention sessions were suspended, and no in-person data collection was conducted. Outcome measures were administered by telephone: participants received paper versions of the questionnaires to follow along while the researcher read the questions aloud and recorded the responses. These procedures ensured continuity of data collection while complying with public health restrictions.

#### Intervention

The MOMANT intervention is a manual-based training and support programme for informal caregivers of people with dementia. The intervention consisted of six structured group sessions (see Table 1 for an overview per session). Together the sessions covered the following core themes: (i) education on dementia, its symptoms, and the healthcare system; (ii) strategies for effective communication with the person with dementia; (iii) skills for managing behavioural changes; (iv) methods on how to cope with caregiver burden; and (v) training on how to engage the person with dementia in activities. The intervention material was developed prior to the study by the research team, based on existing evidence-based approaches to caregiver support [21, 32]. The manual content was finalised in consultation with a focus group consisting of two informal caregivers, one person with dementia, an expert by experience, and healthcare professionals, with the aim to better align the content with the needs of the target group (see also [29]).

**Table 1:** Overview of the sessions of the MOMANT intervention

Session	Topic	Role of the healthcare professional
1	Introduction of the group; information on dementia symptoms and the healthcare system	Lead group introductions and discussion; introduce manual
2	Coping with caregiving; changes in behaviour; practising communication	Lead discussion; provide coping strategies and communication tips
3	How to present activities and motivate the person with dementia	Lead discussion; guide activity selection with caregivers
4	Implementation of activities at home	Lead discussion; encourage caregiver input on home implementation
5	Caregivers’ experience of presenting activities at home	Lead discussion; address questions based on caregivers’ home experience
6	Overall experience with the intervention; revisit previous topics	Lead discussion; support planning for long-term continuation

Sessions 1–3 were held weekly; sessions 4–6 were held every two weeks. Each session lasted approximately 1–1.5 h.

The intervention was person-centred, meaning that while all sessions followed the same structure and topics as outlined in the manual, healthcare professionals encouraged caregivers to share their own experiences and raise questions relevant to their situation, such that the content of the sessions could be tailored to the participating caregivers’ needs and wishes. The shared activities had to be enjoyable and stimulating, physically, cognitively or socially and tailored to the interests and abilities of the person with dementia. The activities were selected jointly by the caregiver and the person with dementia, with guidance from the healthcare professional. The activities could include routine activities (e.g., household tasks), simple physical or cognitive exercises (e.g., going for a walk or reading newspaper headlines together), or new activities introduced during the sessions (e.g., reminiscence activities). A key goal of the intervention was to increase the number of shared activities and the time spent on these activities, whether by intensifying existing activities or engaging in new activities.

The six sessions were delivered over 2 months: the first 3 sessions were held weekly, followed by three sessions every 2 weeks. Each session lasted approximately 1–1.5 h. The intervention was delivered in small groups of caregivers [4–8], led by a local healthcare professional, and took place at the dementia daycare or Meeting Centre. The healthcare professional leading the sessions was staff, a coordinator at a dementia daycare centre or a dementia case manager. The healthcare professional was trained by the research team on how to present the intervention in a single session of 2 to 2.5 h at the site of the healthcare organisation. The training focused on how to present the intervention sessions as outlined in the manual. All professionals delivering the intervention had a background in nursing, social work or care and welfare, and had extensive experience with supporting people with dementia and their informal caregivers.

All participants received the MOMANT manual, which provided an overview of the topics, detailed descriptions of activities and additional resources, such as relevant websites and telephone numbers of available health services. The manual aimed: (i) to minimize preparation time required by healthcare professionals presenting the intervention, and (ii) to provide additional information to the participating caregivers. If a session was missed, participants could consult the manual for the relevant content of that particular session and raise any questions at the start of the subsequent session.

To facilitate implementation of the intervention at home and to assess intervention fidelity, the healthcare professional visited the dyad once at home between the 5th and 6th session of the intervention. By this point, caregivers were already familiar with the intervention content and had begun implementing shared activities with the person with dementia at home. This allowed the healthcare professional to observe how the activities were carried out at home and to assess whether the educational suggestions and advice were being implemented. This visit provided the informal caregivers with the opportunity to ask questions in an individual setting as opposed to the group sessions. Based on this visit, the healthcare professional could then provide tailored guidance on the long-term implementation of the activities.

#### Care as usual

Participants in the control condition continued with treatment as usual. In the Netherlands, common practice regarding dementia care varied between regions, but most community-dwelling persons with dementia were cared for by their spouse or relative, and were assigned a dementia case manager by their general practitioner. Almost all participants attended a daycare facility for one or more days per week. Participants in the control condition were allowed to continue using any health, social or voluntary sector service they were already receiving or chose to start receiving during participation in this study. After completing the study, these participants received the MOMANT manual and were offered the opportunity to participate in the MOMANT intervention if they wished.

### Measures

Questionnaires were administered with both the informal caregiver and the person with dementia present, but the answer of the informal caregiver was seen as leading if answers of the dyad differed. More detailed information on the materials used and their psychometric properties can be found in the previously published study protocol [29].

#### Primary outcome measures

The primary outcome measure for the caregiver was health-related quality-of-life assessed with the self-reported *EQ-5D-5L* instrument [33, 34], including the visual analogue scale (VAS). The descriptive system comprises five dimensions: mobility, self-care, usual activities, pain/discomfort and anxiety/depression, which are scored on a five-point scale. For this measure the index score was used, which provides a single summary number representing the health-related quality-of-life. The index scores were derived by applying utility weights to the responses from a value set for the Dutch adult population [29]. A higher score corresponds to a higher health-related quality of life.

The primary outcome measure for the person with dementia was quality of life assessed with the self-reported *Dementia Quality of Life* scale (DQOL; [35]) comprising of 29 items, ranked on a five-point Likert scale. The DQOL measures five QoL domains: self-esteem, positive affect, negative affect, feeling of belonging and sense of aesthetics. A higher score on the DQOL means a better quality of life on that particular domain.

#### Secondary outcome measures

The *Resource Utilization in Dementia* (RUD; [36]) was used to collect socio-demographic characteristics of the dyad including age, gender, relationship with person with dementia, employment status, and living situation. The RUD was also used to measure healthcare resource utilization among persons with dementia and their caregivers, and time spent on formal and informal care.

The *Short Sense of Competence Questionnaire* (sSCQ; [37]) is a seven-item questionnaire that specifically measures the caregiver’s satisfaction and worries in their role as caregiver. In this shorter version, the items refer to three domains: the satisfaction of the caregiver with the person with dementia as recipient of care; the satisfaction of the caregiver with their own performance as caregiver; and the negative consequences that caring has for the social and personal life of the caregiver. A higher score indicates a higher sense of competence in dealing with the burden of caregiving.

The *Positive Experiences Scale* (POS; [38]) consists of eight items to measure positive caregiving experiences, such as intrinsic satisfaction or social enhancement. For caregivers of persons with dementia, the authors recommend excluding two items (‘because of caregiving the relation with my family and friends has become closer’ and ‘I receive a lot of appreciation for the care I give’), as these are considered less relevant in this caregiving context. A higher score indicates a more positive experience towards caregiving.

The *Center for Epidemiologic Studies – Depression* (CES-D1; [39]) is a brief self-report scale designed to measure current self-reported symptoms associated with depression experienced in the past week. It consists of 20 items comprising six domains of depression: depressed mood, feelings of guilt and worthlessness, feelings of helplessness and hopelessness, psychomotor retardation, loss of appetite, and sleep disturbance. For the analyses, the four subdomains were used from the original study: (i) depressed affect, (ii) positive affect, (iii) somatic symptoms, and (iv) interpersonal symptoms [39]. Item 8 (‘I felt hopeful about the future’) was not asked, because it was considered not applicable to and potentially inappropriate for informal caregivers of people with dementia. In earlier research in elderly population, it was also found no to fit the four-factor structure of the instruments [40]. A higher score on the CES-D1 means the presence of more depressive symptoms.

The *Pleasant Events Schedule-Alzheimer’s Disease – Short Form* (PES-AD; [41]) is a commonly used self-report assessment that attempts to identify activities that people with dementia may find enjoyable. It was used to assess the overall frequency of engagement in 20 activities by the person with dementia, as reported by the caregiver and person with dementia together. A higher score indicates that the person with dementia was both engaged in and also enjoyed more activities.

*Interview for Deterioration in Daily Living Activities in Dementia* (IDDD; [42]), which consists of 33 items, reflecting the initiative to perform and actual performance of self-care and more complex activities, as reported by the caregiver. The IDDD consists of two scales: one related to basic activities of daily living (ADL), and one related to instrumental activities of daily living (IADL). A higher score on this measure indicates more cognitive impairment and thus a higher dependency on the caregiver in daily life.

The *Global Deterioration Scale* (GDS; [43]) was used to assess presence and severity of the dementia. The GDS identifies seven stages of severity (1 no cognitive impairment – 7 very severe cognitive impairment) based on the described symptoms of cognitive decline in these stages. The GDS was completed in a structured interview with the caregiver.

#### Treatment adherence

To assess whether the healthcare professionals presented the intervention as intended, a sample of sessions from the intervention were video recorded with permission from the healthcare professional and the participating caregivers in this session. The recordings were scored against a list of predetermined criteria by two independent raters (SB and MM) from the research team. The criteria included use of the manual and adherence to the topics covered in the manual as well as adherence to the guidelines on leading and presenting the sessions outlined in the train-the-trainer session. To assess whether the informal caregivers followed the intervention instructions and carried out the activities with the person with dementia as intended, the implementation of the intervention and the activities were scored during a home visit by the healthcare professional, with permission of the dyad.

#### Subjective evaluation

Healthcare professionals and informal caregivers allocated to the intervention condition were asked to evaluate the intervention. The healthcare professionals were asked to evaluate the training that they had received beforehand and the content of the intervention through a questionnaire, to be returned anonymously. At both follow up measurements, a questionnaire for the informal caregivers was left behind to evaluate the manual and the intervention so that it could be completed at home, and returned anonymously. These questionnaires were specifically developed for this study, and an English translation of these questionnaires can be found in Supplementary Files 2, 3 and 4, including (a) a 3-month evaluation for the intervention group participants; (b) a 6-month evaluation for the intervention group participants; and (c) an evaluation for the healthcare professionals who led the intervention sessions.

#### Time of admission to residential care

All informal caregivers who had completed the 6 month follow up were contacted by telephone 18 to 24 months after the final assessment and asked about the current (living) situation of the person with dementia: (i) whether they were still residing at home with no significant changes; (ii) whether they were still living at home but required additional (formal) care due to increased cognitive or behavioural changes in the person with dementia; (iii) whether the person with dementia had been admitted to residential care; or (iv) whether the person with dementia had passed away.

### Data analysis

Sample characteristics were obtained using descriptive analyses. As randomisation occurred at the level of the centre, we inspected potential differences between the control and intervention group at baseline using Chi-Square tests or either independent-samples t-tests or Mann-Whitney U tests as appropriate.

Linear mixed effects models were used to examine the effect of the intervention on the outcome measures, according to the intention-to-treat principle. Each model included fixed effects for group (dummy variable; intervention vs. control), time (dummy variable; 6-month follow-up vs. 3-month follow-up) and the time by group interaction. All models were adjusted for the baseline value of the respective outcome measure in addition to any characteristics on which the intervention and control groups differed significantly (see Results section). All models included random intercepts. Estimated marginal means were derived from these models and compared to investigate differences between groups at the 3- and 6-month follow-up. Significance level was set to 0.05. A power analysis showed that at least 64 dyads in each group were required to achieve 80% power to detect significant differences between groups on the primary outcome measures assuming a medium effect size (Cohen’s d = 0.5; [29]). The intention-to-treat analysis was followed by three sensitivity analyses examining the effect of the intervention in specific subsamples; (1) the subsample of participants who had completed all intervention sessions, to control for variation in treatment adherence; (2) the subsample who were recruited through organisations other than Meeting Centres, to avoid possible contamination from the support programme offered at Meeting Centres; and (3) the subsample with milder dementia (GDS = < 4), to examine the possibility that the intervention was more effective when presented in the earlier stages of dementia.

The data collected for the process evaluation was analysed using descriptive statistics.

Analyses were performed in Rstudio 4.4.0 using packages *nlme* and *emmeans.*

## Results

### Sample Characteristics

**Fig. 1:**
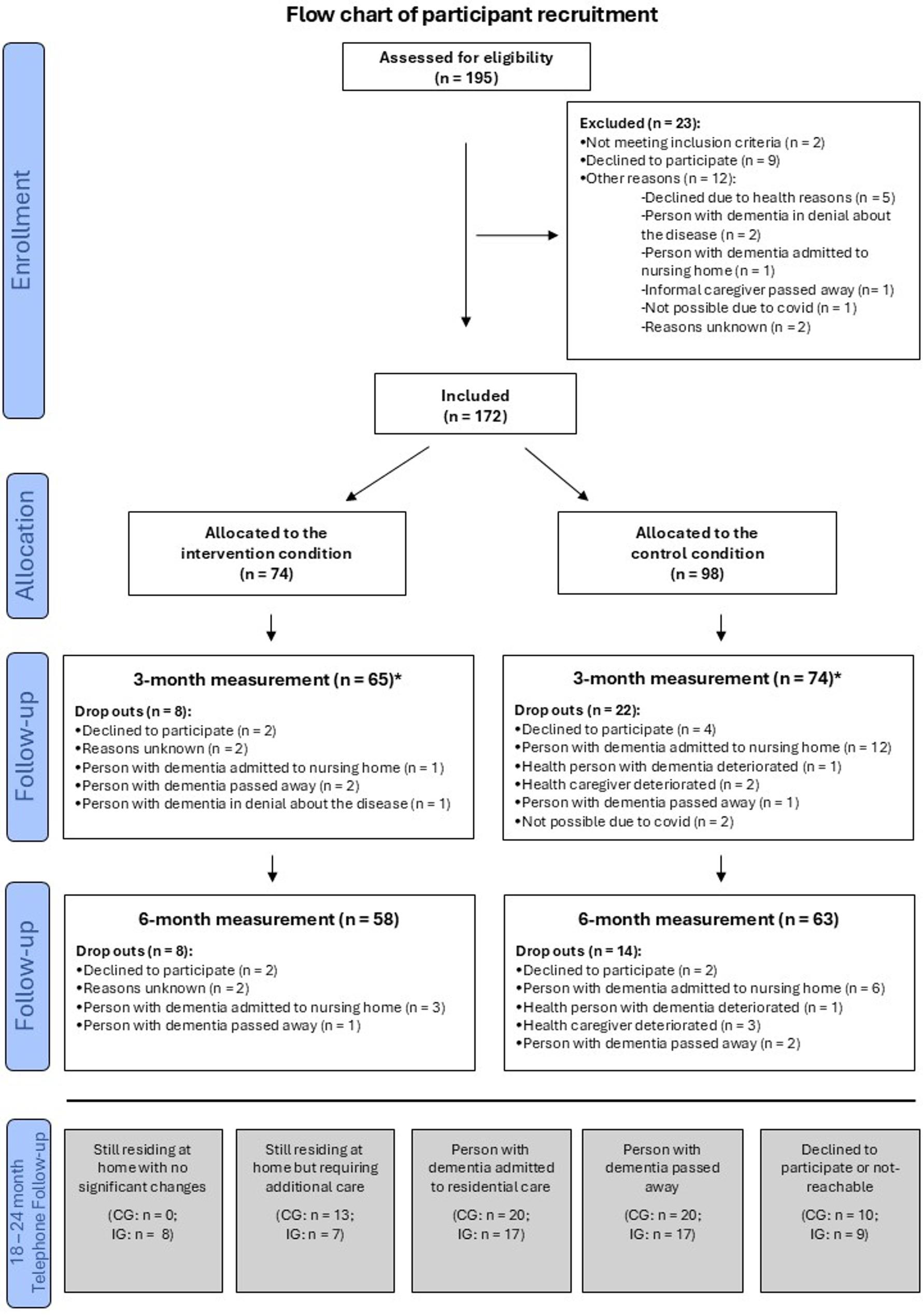
Flow chart of participant recruitment and contact after final assessment. Note * indicates that some caregivers did notparticipate in the 3-month measurement but did participate in the 6-month measurement. CG = control group, IG = intervention group

Characteristics of the sample can be found in Table 2. The overall sample consisted of 172 dyads recruited by 56 different organisations across the Netherlands. Of this sample 74 dyads (43%) were assigned to the intervention condition; and 98 dyads (57%) to the control condition. A flowchart of the progress of the MOMANT study regarding participant recruitment and telephone contact after the final assessment can be found in Figure 1.

**Table 2:** Characterisetics of the sample

Characteristic	MOMANT Intervention (*n* = 74)		Control (*n* = 98)		*p*	
	Informal caregivers	Persons with dementia	Informal caregivers	Persons with dementia	Informal caregivers	Persons with dementia
**Gender (female)**	54 (73%)	26 (35%)	69 (70%)	49 (50%)	.71	.05
**Age (in years)**	69.9 (9.4) [45–94]	78.3 (7.3) [61–93]	69.4 (12.1) [65–98]	82.1 (6.7)* [45–93]	.79	**< .01**
**Educational level**					.32	.21
Low education	12 (16%)	24 (33%)	11 (11%)	34 (34%)		
Mid education	39 (53%)	39 (52%)	55 (56%)	39 (40%)		
High education	23 (31%)	11 (15%)	32 (33%)	25 (26%)		
**Relationship**					.14	
Spouse	55 (74%)		57 (58%)			
Child	16 (22%)		36 (37%)			
Friend	2 (3%)		2 (2%)			
Other	1 (1%)		3 (3%)			
**Cohabiting**	57 (77%)		62 (63%)		.053	
**Living area**					.51	
Rural area	31 (42%)		46 (47%)			
Urban area	43 (58%)		52 (53%)			
**Informal care (daily hours) –** **Provided by caregiver**	4.69 (4.70)		6.2 (7.75)		.42	
Type of dementia*						
Alzheimer’s disease		34*		30		**.04**
Vascular dementia		16		23		.77
Frontotemporal dementia		2		5		.70
Lewy body dementia		4		3		.47
Mild Cognitive Impairment (MCI)		0		7*		**.02**
Other		5		8		.73
Unknown at time of assessment		21		27		.56
**Global Deterioration Scale**						.79
GDS 2: Mild Cognitive Impairment		16 (22%)		18 (18%)		
GDS 3: Mild Dementia		20 (27%)		24 (25%)		
GDS 4: Moderate Dementia		32 (43%)		44 (45%)		
GDS 5: Moderately Severe Dementia		6 (8%)		12 (12%)		
**Time since diagnosis (in months)**		27.8 (28.3)		38.4 (32.1)*		**.03**

Data depicted as n(%), mean (standard deviation) or [range]. For analyses requiring duration of dementia diagnosis, the date that the person with dementia began attending a dementia daycare centre was used as proxy for date of diagnosis, as a formal diagnosis is required to attend such centres

Of the overall sample, the majority of the informal caregivers was female (*n* = 123, 72%) and had attended secondary education (*n* = 94, 55%). The average age was 69.6 (SD:11.0, range 45–98) years for the caregivers and 80.5 (SD:7.2, range 45–93) years for the persons with dementia. Most dyads were spouses (65%, *n* = 112), followed by adult-children (31%, *n* = 52). Most persons with dementia were diagnosed with Alzheimer’s disease (*n* = 65, 38%) and scored in the moderate dementia range on the Global Deterioration Scale (*n* = 76, 44%). Persons with dementia received a daily average of 5.56 hours (SD:6.49) of informal care from their primary caregiver.

At baseline, the groups differed significantly on the following characteristics: age of the person with dementia (intervention: 78.3 years (SD:7.3), control: 82.1 years (SD:6.7), t = 3.56, *p* < .01) as well as time since diagnosis (intervention: 28 months (SD:28), control: 38 months (SD:32), t = 2.28, *p* = .02). Additionally, the intervention group had more persons diagnosed with Alzheimer’s disease (χ^2^ = 4.24, *p* = .04), and the control group had more persons diagnosed with Mild Cognitive Impairment (Fisher’s exact test, *p* = .02). The intervention group also contained more individuals recruited from daycare setting (versus Meeting Centre or similar support programme) than the control group (χ^2^ = 4.65, *p* = .03). See Table 3 for an overview of the baseline outcome measures per group.

**Table 3:** Mean, standard deviation, and range of the self-reported measures of the sample at baseline

Self-reported measure	Baseline			
Overall sample (*N* = 172)	Intervention (*n* = 74)		Control (*n* = 98)	
*Measures for the informal caregiver*				
** Quality of life (EQ-5D-5L)**	0.86 (0.2)	[0–1]	0.82 (0.2)	[0–1]
** Overall health (VAS score)**	77.1 (10.9)	[50–100]	74.2 (16.0)	[15–100]
** Depressive symptomatology (CES-D)**	7.7 (18.3)	[0–29]	8.7 (17.0)	[0–44]
Positive affect	2.0 (1.9)	[0–8]	2.4 (2.4)	[0–9]
Depressed affect	2.6 (2.7)	[0–12]	2.5 (2.7)	[0–12]
Somatic symptoms	4.7 (3.8)	[0–18]	4.9 (3.4)	[0–16]
Interpersonal symptoms	0.4 (0.8)	[0–4]	0.3 (0.8)	[0–4]
** Sense of Competence (SCQ)**	21.0 (4.4)	[7–28]	20.7 (4.6)	[7–28]
Consequences for personal life	5.7 (2.1)	[2–10]	5.1 (2.0)	[2–10]
Satisfaction as caregiver	7.1 (2.4)	[2–10]	7.5 (2.4)	[2–10]
Satisfaction with care recipient	8.3 (1.4)	[3–9]	8.1 (1.5)	[3–9]
** Positive Experiences Scale (POS)**	12.8 (4.6)	[6–26]	12.2 (5.0)	[6–26]
*Measures for the person with dementia*				
** Dementia quality of life (DQOL)**				
Positive affect	21.5 (3.8)	[6–27]	21.0 (4.0)	[9–28]
Negative effect	25.3 (5.6)	[13–37]	26.3 (6.0)	[15–36]
Sense of belonging	11.0 (1.8)	[7–15]	10.6 (2.2)	[4–15]
Aesthetics	18.1 (3.5)	[10–24]	18.3 (3.9)	[5–25]
Self esteem	13.7 (2.5)	[7–18]	12.9 (2.9)	[6–19]
** Pleasant Events Schedule (PES)**				
Frequency	41.6 (6.0)	[25–54]	41.0 (6.0)	[27–54]
Enjoyment	51.7 (4.6)	[41–59]	51.7 (5.4)	[33–60]
** Interview for Deterioration in Daily Living Activities in Dementia (IDDD)**				
Activities of Daily Living	22.4 (7.5)	[7–49]	23.1 (8.5)	[7–48]
Instrumental Activities of Daily Living	26.6 (7.4)	[13–48]	28.1 (7.8)	[10–48]
** Global Deterioration Scale (GDS)**	4.4 (0.9)	[3–6]	4.5 (0.9)	[3–6]

Data depicted as mean (standard deviation) and [range] per group. The total range for the outcome measures is as follows EQ-5D-5L: [0–1]; VAS: [0-100]; CES-D: [0–57]; SCQ: [7–29]; POS: [6–26]; DQOL, positive affect: [6–30], negative effect: [11–55], sense of belonging: [3–15], aesthetics: [5–25], self-esteem: [4–20]; PES: [0–60]; IDDD: [0–51]; GDS: [0–7]. VAS = Visual analogue scale, CES-D = Center for Epidemiologic Studies Depression scale

Regarding treatment adherence, caregivers in the intervention group on average attended 4.9 (SD:1.3) out of 6 sessions. In the home visits, the healthcare professionals rated the dyads on how the activities were carried out at home with an average of 6.5 (SD: 1.9, *n* = 51) out of 10, and with an average of 6.1 (SD:2.3, *n* = 51) out of 10 on how the educational suggestions and advice were being implemented at home.

### Intervention effects

The following variables were adopted as covariates in the linear mixed models to adjust for baseline differences between the intervention and control group: time since diagnosis (in months), age of the person with dementia (in years), diagnosis of Alzheimer’s disease, diagnosis of Mild Cognitive Impairment and setting (daycare centre versus Meeting Centre or similar support programme). The intervention effects are shown in Table 4. Estimated means based on the models at 3- and 6-months are shown in Table 5. Fixed estimates of the covariates can be found in Supplementary File 3.

**Table 4:** Treatment group interaction effects of linear mixed models for each outcome measure and group differences at 3- and 6-months follow-up derived from these models

Outcome measure	Group*time			Mean Differences*			
	B(SE)	95%CI	*p*	3 months	*p*	6 months	*p*
*Measures for the informal caregiver*							
** Quality of life (EQ-5D-5L)**	0.00(0.02)	[-0.04;0.03]	.89	0.02(0.02)	.45	0.01(0.02)	.52
** Overall health (VAS)**	-4.26(2.25)	[-8.71;0.18]	.06	2.24(2.14)	.30	-2.02(2.23)	.37
** Depressive symptomatology (CES-D)**							
* Total score*	2.22(3.90)	[-5.50;9.95]	.57	2.89(2.91)	.32	5.11(3.06)	.10
* Positive affect*	0.31(0.31)	[-0.31;0.92]	.33	0.15(0.27)	.60	0.45(0.29)	.12
* Depressed affect*	0.42(0.44)	[-0.45;1.29]	.34	0.09(0.38)	.81	0.51(0.40)	.19
* Somatic symptoms*	0.38(0.60)	[-0.37;1.98]	.53	-0.43(0.57)	.45	0.80(0.59)	.18
* Interpersonal symptoms*	0.13(0.17)	[-0.21;0.48]	.43	-0.06(0.15)	.68	0.08(0.15)	.62
** Sense of competence (SCQ)**							
* Total score*	-0.25(0.63)	[-1.49;0.99]	.69	0.19(0.61)	.76	-0.06(0.64)	.92
* Consequences for personal life*	-0.40(0.37)	[-1.12;0.33]	.28	0.32(0.33)	.33	0.07(0.34)	.84
* Satisfaction as caregiver*	-0.08(0.36)	[-0.79;0.63]	.82	0.20(0.31)	.52	0.12(0.33)	.72
* Satisfaction with care recipient*	0.25(0.23)	[-0.21;0.72]	.27	-0.36(0.12)	.13	-0.10(0.24)	.67
** Positive Experience Scale (POS)**	**-0.51(0.24)**	**[-0.99; -0.04]**	**.03**	**0.52(0.22)**	**.02**	0.00(0.23)	.98
*Measures for the person with dementia*							
** Quality of Life (DQOL)**							
* Positive affect*	-1.39(0.85)	[-3.10;0.30]	.05	-0.01(0.68)	.99	-1.4(0.74)	.06
* Negative affect*	0.14(1.39)	[-2.64;2.92]	.11	-0.17(1.20)	.89	-0.03(1.32)	.98
* Sense of belonging*	-0.67(0.46)	[-1.61;0.26]	.92	0.29(0.37)	.44	-0.38(0.41)	.36
* Aesthetics*	0.50(0.81)	[-1.21;2.11]	.16	-0.07(0.68)	.92	0.43(0.74)	57
* Self esteem*	-1.39(0.70)	[-2.80;0.01]	.54	0.80(0.52)	.13	-0.59(0.58)	.31
** Pleasant Events Schedule (PES)**							
* Frequency*	**-1.98(0.83)**	**[-3.62;-0.33]**	**.02**	**2.00(0.91)**	**.03**	0.02(0.94)	.98
* Enjoyment*	0.08(0.92)	[-1.47;1.64]	.08	0.21(0.86)	.81	0.29(0.87)	.74
** IDDD**							
* Activities of Daily Living*	2.31(1.35)	[-0.36;4.99]	.09	-2.27(1.23)	.07	0.04(1.28)	.98
* Instrumental Activities of Daily Living*	1.40(1.47)	[-1.51;4.31]	.34	-2.23(1.42)	.12	-0.83(1.48)	.58

Models were adjusted for baseline score on the respective outcome measure and covariates. *Mean differences are calculated as intervention minus control, and displayed with the standard error. Significant effects are indicated with **bold** fontNote. VAS = visual analogue scale, CES-D = Center for Epidemiologic Studies Depression scale, DQOL = DementiaQuality of Life, IDDD = Interview for Deterioration in Daily Living Activities in Dementia

**Table 5:** Estimated means of outcome measures derived from linear mixed model for control and intervention group at 3- and 6-months follow-up

Outcome measure	3 months		6 months	
	Intervention	Control	Intervention	Control
*Measures for the informal caregiver*				
**Quality of life (EQ-5D-5L)**	0.92 [0.86–0.97]	0.90 [0.85–0.95]	0.90 [0.84–0.95]	0.89 [0.84–0.93]
** Overall health (VAS)**	76.4 [71.4–81.4]	74.1 [69.7–78.6]	72.0 [66.9–77.1]	74.0 [69.4–78.6]
** Depressive symptomatology (CES-D)**				
* Total score*	13.6 [7.31–19.9]	10.7 [5.09–16.4]	14.5 [8.05–21.0]	9.4 [3.54–15.3]
* Positive affect*	2.27 [1.64–2.90]	2.12 [1.57–2.68]	2.62 [1.98–3.26]	2.17 [1.60–2.75]
* Depressed affect*	3.09 [2.24–3.93]	3.00 [2.27–3.75]	3.10 [2.23–3.96]	2.58 [1.80–3.36]
* Somatic symptoms*	5.22 [3.89–6.55]	5.64 [4.47–6.82]	5.42 [4.07–6.77]	5.04 [3.84–6.25]
* Interpersonal symptoms*	0.41 [0.08–0.74]	0.47 [0.18–0.76]	0.63 [0.30–0.97]	0.56 [0.25–0.86]
** Sense of competence (SCQ)**				
* Total score*	21.3 [19.9–22.8]	21.2 [19.9–22.4]	20.7 [19.3–22.1]	20.8 [19.5–22.1]
* Consequences for personal life*	5.39 [4.62–6.16]	5.06 [4.38–5.75]	5.08 [4.30–5.86]	5.15 [4.45–5.86]
* Satisfaction as caregiver*	7.75 [7.04–8.46]	7.55 [6.91–8.18]	7.54 [ 6.82–8.27]	7.42 [6.77–8.08]
* Satisfaction with care recipient*	8.22 [7.67–8.77]	8.58 [8.09–9.06]	8.10 [ 7.55–8.66]	8.21 [7.71–8.70]
** Positive Experience Scale (POS)**	**3.99 [3.49–4.50]**	**3.47 [3.02–3.92]**	3.65 [3.14–4.16]	3.64 [3.19–4.10]
*Measures for the person with dementia*				
** Quality of Life (DQOL)**				
* Positive affect*	19.6 [18.1–21.1]	19.6 [18.3–20.9]	19.0 [17.4–20.6]	20.4 [19.0–21.8]
* Negative affect*	25.1 [22.1–28.0]	25.2 [22.6–27.9]	25.7 [22.6–28.7]	25.7 [22.8–28.6]
* Sense of belonging*	11.3 [10.4–12.1]	11.0 [10.3–11.7]	11.0 [10.1–11.8]	11.3 [10.5–12.1]
* Aesthetics*	16.5 [14.9–18.2]	16.6 [15.1–18.1]	17.2 [15.4–18.9]	16.8 [15.1–18.4]
* Self esteem*	14.2 [13.0–15.4]	13.4 [12.3–14.5]	13.9 [12.6–15.2]	14.5 [13.3–15.7]
** Pleasant Event Schedule (PES)**				
* Frequency*	**42.5 [40.4–44.7]**	**40.5 [38.6–42.4]**	41.1 [39.0–43.2]	41.1 [39.1–43.0]
* Enjoyment*	51.0 [49.0–53.0]	50.8 [49.0–52.5]	51.6 [49.6–53.6]	51.3 [49.6–53.1]
** IDDD**				
* Activities of Daily Living*	23.3 [20.5–26.1]	25.6 [23.1–28.1]	23.6 [20.7–26.5]	23.5 [20.9–26.1]
* Instrumental Activities of Daily Living*	26.0 [22.7–29.4]	28.3 [25.3–31.2]	27.8 [24.4–31.2]	28.6 [25.6–31.7]

Estimated means with [95% CI] are provided. Models were adjusted for baseline score on the respective outcome measure and covariates. Significant differences are indicated in bold (also see Table 4)Note. VAS = visual analogue scale, CES-D = Center for Epidemiologic Studies Depression scale, DQOL = DementiaQuality of Life, IDDD = Interview for Deterioration in Daily Living Activities in Dementia

### Primary outcomes

#### Health-related quality of life of the caregiver (EQ-5D-5L and EQ-VAS)

There was no significant interaction between group and time for the EQ-5D-5L index score or the EQ-VAS score, which indicated no significant effect of the intervention on health-related quality of life of the caregiver over time. The separate effects at 3 and 6 months were also not significantly different between the groups. The distribution of the scores across the five levels of each dimension can be found in Supplementary File 4.

#### Quality of life of the person with dementia (DQOL)

There was no significant interaction between group and time on the DQOL subscales, indicating no evidence of effect of the intervention on quality of life of the person with dementia. The separate effects at 3 and 6 months were also not significantly different between the groups on all DQOL subscales.

### Secondary outcomes

#### Shared activities (Pleasant Events Schedule; Frequency and Enjoyment)

**Fig. 2:**
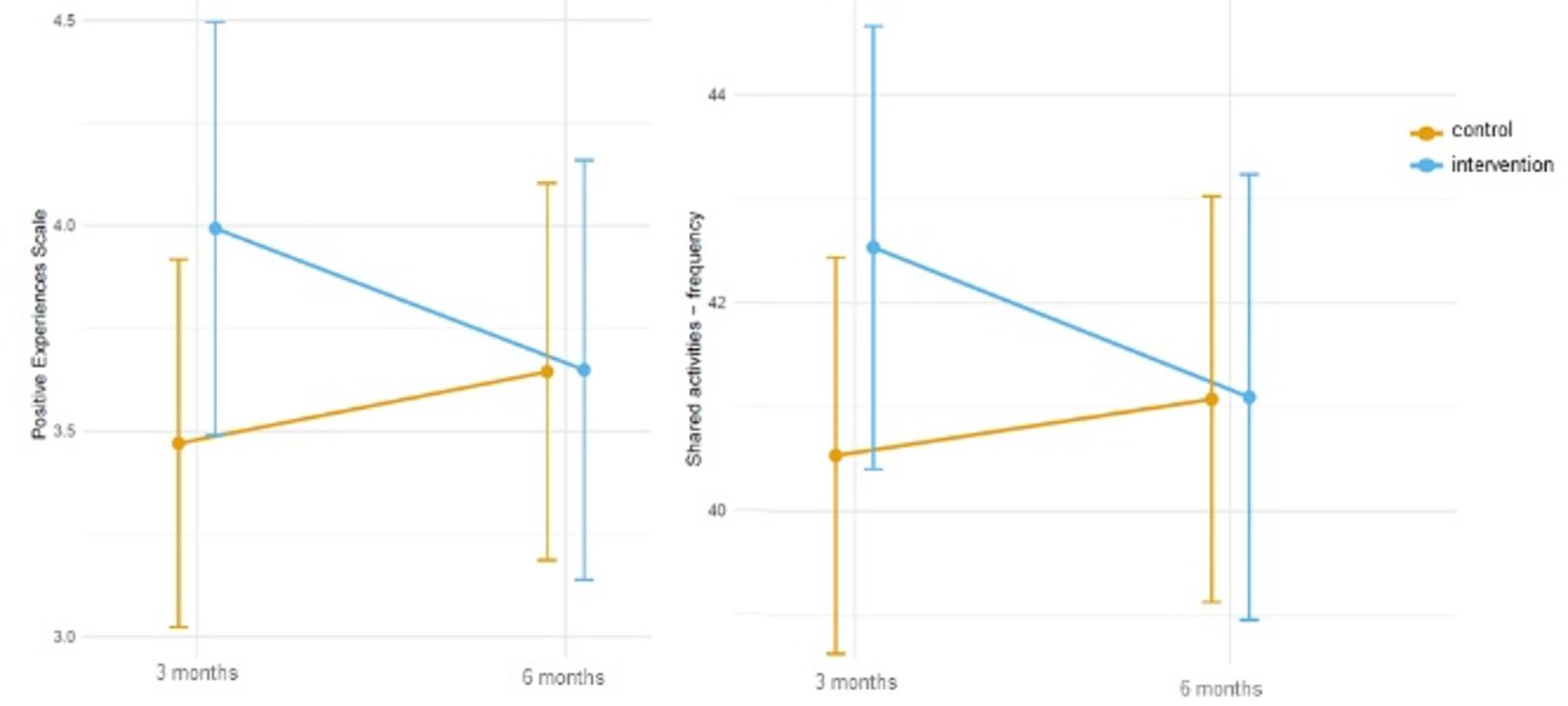
Estimated marginal means of Positive Experiences Scale and Shared events (frequencydimension) scores by group and at 3 and 6 months Model-adjusted means with 95% CI's are shown. Estimates are derived from the linear mixed-effects model controlling for baselinescore and covariates. Higher scores indicate more positive caregiving experiences and a higher frequency of shared activities, respectively

There was a significant interaction between group and time for the Frequency dimension of the Pleasant Events Schedule (B = -1.978, SE = 0.83, t = -2.38, *p* = .02), indicating that the difference in the frequency of activities decreased between groups over time (see Figure 2 for the model-adjusted means and Supplementary File 5 for raw scores over time of the individual participants). At 3 months, the intervention group reported a higher frequency of joint activities compared to the control group (mean difference = 1.997, SE = 0.91, pple*p* = .03). At 6 months, reported frequency of joint activities were similar between groups (mean difference = 0.02, SE = 0.91, *p* = .98). There was no significant interaction between group and time for the Enjoyment dimension of the Pleasant Events Schedule, indicating no evidence of an effect of the intervention over time.

#### Positive caregiving experiences (POsitive experiences Scale)

There was a significant interaction between group and time (B = -0.52, SE = 0.24, t = -2.16, *p* = .03) for the Positive experiences scale scores, indicating different trajectories in positive caregiving experiences between the intervention and control group over time (see Fig. 2). At 3 months, the intervention group had a significantly higher score than the control group (mean difference = -0.52, SE = 0.22, *p* = .019). At 6 months, scores were similar between groups (mean difference = 0.00, SE = 0.23, *p* = .984).

#### Sense of competence of the caregiver (SCQ)

There was no significant interaction between group and time for either the total Sense of Competence score or its subscales, indicating there was no evidence that the intervention affected the sense of competence of the caregiver over time. The separate effects at 3 and 6 months were also not significantly different between the groups.

#### Depressive symptomatology of the caregiver (CES-D)

There was no significant interaction between group and time for any of the CES-D measures, indicating no evidence of a significant effect of the intervention on dimensions of depressive symptomatology over time. Likewise, the separate effects at 3 and 6 months showed no significant differences between the intervention and control group.

#### ADL and IADL dependency (IDDD)

There was no significant interaction between group and time on ADL dependency or [I]ADL dependency, indicating no evidence of effect of the intervention on the degree of dependency of the person with dementia for basic and instrumental activities of daily living. Although the model indicated a slight difference regarding ADL dependence at 3 months between the intervention group (estimated marginal mean = 23.3) and control group (estimated marginal mean = 25.6), this difference did not reach statistical significance (mean difference = 2.27, SE = 1.23, t = 1.85, *p* = .07).

### Covariates

When looking at the covariates, the baseline score was positively related to the follow-up scores (*p*’s < .05) for every measure except the CES-D total score. A diagnosis of MCI was negatively related to the DQOL Positive subscale (B = -3.14, SE = 1.17, t = -2.69, *p* = .01). A diagnosis of Alzheimer’s disease was positively related to the SCQ subscale Satisfaction of the caregiver with their own performance as caregiver (B = 0.80, SE = 0.26, t = 3.10, *p* < .001) and to the CES-D subscale Interpersonal symptoms subscale (B = 0.27, SE = 0.12, t = 2.26, *p* = .03). Daycare centre setting (as opposed to a Meeting Centre or a similar support programme) was negatively related to the CES-D subscale Positive affect (B = -0.51, SE = 0.22, t = -2.28, *p* = .02). Time itself was negatively related to the SCQ subscale Satisfaction with the person with dementia as care recipient (B = -0.37, SE = 0.16, t = -2.31 *p* = .02) and positively related to the DQOL Self Esteem subscale (B = 1.12, SE = 0.50, t = 2.22, *p* = .03).

### Sensitivity analyses

A sensitivity analysis performed in the subsample of participants that completed all intervention sessions (*n* = 129; *n* = 32 intervention vs. all controls) showed no evidence for an effect of the intervention on most measures. As in the original intention-to-treat analyses, there was a significant interaction between group and time on the frequency dimensions of the Pleasant Events Schedule (B = -2.30, SE = 1.05, t = -2.20, *p* = .03), indicating that the intervention group reported engaging in more shared activities at the 3 months follow-up than the control group. However, the differences in scores at the 3- and 6-months follow-up were not significant (mean difference at 3 months = -1.89, mean difference at 6 months = 0.42, p’s > .05). There was also a significant effect of the intervention on the CES-D subscale, interpersonal dimension (B = 0.61, SE = 0.22, t = 2.82, *p* < .01). At 3 months, scores were similar between groups (mean difference = 1.45, SE = 0.18, *p* = .43), but at 6 months the intervention group had a significantly higher score, indicating more interpersonal difficulties than the control group (mean difference = -0.46, SE = 0.20, *p* = .02). There was no significant effect for the Positive experiences scale (*p* = .08), even though this was present in the original intention-to-treat analyses. A second sensitivity analysis was performed in the subsample of participants who were recruited from centres other than Meeting Centres (*n* = 98; *n* = 46 intervention vs. *n* = 52 control). The results revealed no significant intervention effects on any of the outcome measures and trends (.05 < *p* < .10) for the measures that were significant in the original analysis (Pleasant Events Schedule and Positive Experience Scale). A third sensitivity analysis in the subsample of participants in milder dementia stages (GDS score of 4 or lower; *n* = 78; *n* = 36 intervention vs. *n* = 42 control), revealed results comparable to the original analyses. There was an effect of intervention on the Positive Experience Scale with a similar magnitude and direction as in the original analysis (B = -0.85, SE = 0.35, t = -2.45, *p* = .02). The effect on the Pleasant Events Schedule was no longer significant.

### Subjective evaluations of the intervention

Healthcare professionals and informal caregivers evaluated the MOMANT intervention in a very positive way. Shortly after completing the intervention, healthcare professionals in the intervention group (*N* = 15) were invited to complete a brief questionnaire. Almost all professionals (87%, *n* = 13) considered the intervention a valuable addition to their work, and 93% (*n* = 14) expected to continue using the intervention or parts of it in the future. Most found the training they received beforehand to be clear and sufficient (87%, *n* = 13). All professionals appreciated the person-centred approach in leading the sessions, although a few (20%, *n* = 3) indicated they would have preferred more structure. The language of the manual was deemed appropriate for informal caregivers, and all found the informational section (covering the psychoeducation component) useful. Opinions on the activity suggestions in the manual were more mixed but most professionals found them somewhat useful (53%, *n* = 8) or largely useful (40%, *n* = 6). Similarly, opinions on the added value of the home visit varied, but most (60%, *n* = 9) considered it useful to visit the dyad individually, and the majority (71%, *n* = 10) believed that the informal caregiver also found the visit beneficial.

In addition to the perspectives of healthcare professionals, informal caregivers were also asked to evaluate their experiences with the intervention. At the first follow-up at three months, 40 out of 65 (62%) informal caregivers responded. Most (83%, *n* = 33) found the sessions useful overall, but two aspects were highlighted as particularly valuable: 1. the information on dementia and communication strategies, which many noted would have been especially useful shortly after diagnosis, and 2. the opportunity for peer contact in a small group setting, which facilitated personal support and closer connections with the healthcare professional. Nearly all caregivers (93%, *n* = 37) reported having learned something new and felt that the information was generally clear and complete. Suggestions for activities were also seen as at least somewhat useful, and the majority (53%) of caregivers reported that they used them at least once a week or more. Most caregivers (85%, *n* = 34) believed the activities positively affected the person with dementia, however, a small minority (10%, *n* = 4) reported negative effects, such as causing stress or confusion in the person with dementia and found it burdensome or challenging to implement the activities at home. Nonetheless, the majority (60%, *n* = 24) indicated they were likely to continue using parts of the intervention. Many caregivers also described a shift in how they related to the person with dementia, reporting increased patience, understanding, and an improved interaction. At the second follow-up at six months, 34 informal caregivers (59%) responded. Most (74%, *n* = 25) reported still using the manual. Nearly all (97%, *n* = 33) continued to engage in shared activities, and the majority (74%, *n* = 25) still used suggestions from the manual, indicating ongoing positive effects on dyadic interactions. Many caregivers also indicated that they continued to see other caregivers from their group after completing the intervention. Nearly all caregivers (97%, *n* = 33) stated they would recommend the intervention to others.

## Discussion

### Summary and interpretation

This study evaluated the effectiveness of MOMANT, a caregiver-led psychosocial intervention that combined psychoeducation and training informal caregivers to engage the person with dementia in stimulating activities at home. The intervention was found to be feasible and acceptable by healthcare professionals and caregivers. No significant effect was found on the primary outcomes, i.e., quality of life of the informal caregiver and the person with dementia, or on secondary outcome measures such as depressive symptomatology, sense of competence, overall self-rated health or [I]ADL dependency. However, the intervention group did report a significantly higher frequency of shared activities and had a more positive caregiving experience compared to the control group immediately after the intervention.

Participants in the intervention group engaged significantly more in shared pleasant activities than those in the control group, shortly after the intervention, although this effect was not sustained over time. This suggests that the caregivers successfully applied the intervention strategies to engage the person with dementia in stimulating activities, and that caregivers were able to apply what they had learned in the intervention at home, but the effect was not maintained three months later. These findings are in line with previous studies on activity-based interventions that aimed to promote engagement and meaningful interaction in the home setting [9, 24], as well as other studies that found that a caregiver-led approach was feasible [21, 44]. Earlier research has found that interventions work best when they are person-centred, adapting activities to the interests and abilities of the person with dementia and chosen together with the caregiver [7, 45]. This might explain why most caregivers who evaluated the MOMANT intervention indicated that they continued the activities after the study period had ended. As the disease progresses, maintaining opportunities for shared pleasant activities may help alleviate caregiver burden, as well as help strengthen the bond within the dyad [14, 46]. Supporting and preserving emotional connection between the caregiver and care recipient should be considered a key goal of any intervention [46]. MOMANT demonstrated that meaningful activities can be stimulated at home, with the potential to enhance relational quality and promote continued activity beyond the intervention period.

Caregivers in the intervention group reported significantly more positive caregiving experiences compared to the control group at the 3-month follow-up, but this effect was not maintained at follow-up. The Positive Experience Scale used in this study includes items that capture aspects such as forming new social connections through caregiving, acquiring new knowledge and skills, and enjoying meaningful moments with the person with dementia [47]. These items align closely with the content of the MOMANT intervention and its emphasis on education, peer support, and promoting engagement in shared activities of the caregiver with the person with dementia. A growing body of research has pointed out that positive aspects of caring can act as protective factors against caregiver burden and stress [48, 49], and a more positive caregiving experience has been associated with improved caregiver well-being [15, 50].

The finding that effects of the intervention were observed at the 3-month but not at the 6-month follow-up suggests that the benefits may not be sustained without ongoing support, which is not uncommon in the psychosocial intervention literature [24, 45]. Maintaining gains achieved through the intervention likely requires continued practice and encouragement from healthcare professionals or peers. As dementia is a progressive disease, the challenges faced by caregivers and persons with dementia change over time, which may make it increasingly difficult to sustain the effects of a time-limited intervention. Notably, although the intention-to-treat analyses showed no significant effect on the CES-D interpersonal symptoms dimension, the sensitivity analysis among those who attended all intervention sessions revealed a significant effect at 6 months, with higher scores indicating more interpersonal distress in the intervention group. A possible explanation is that the group sessions could have provided a source of social connection that was no longer available after the intervention ended, which may have contributed to increased feelings of loneliness or social isolation at 6 months. However, this finding should be interpreted with caution given the risk of selection bias in completer analyses and the small intervention sample (*n* = 32).

There are several possible explanations for neither finding effects on the measures for quality of life of the person with dementia or the informal caregiver, nor on other secondary outcome measures such as caregivers’ depressive symptomatology, sense of competence and overall self-rated health, or the person with dementia’s [I]ADL dependency, despite a sample size large enough for sufficient statistical power. Ceiling effects may in part explain these findings. The caregiver scores at baseline for both quality of life (EQ-5D-5L) and sense of competence (SCQ) were relatively high, restricting the potential scope for improvement. Given that a higher sense of competence has been generally linked to better mental health and fewer depressive symptoms [51], this may also explain the relatively low baseline levels of depressive symptomatology in this sample. Outcome measures with a broader score range, or those more sensitive to subtle change, may better capture intervention effects in this population [52]. In addition, given that most persons with dementia in this study had received their diagnosis two to four years prior to enrolment and were at a moderate to moderately severe stage of the disease, the intervention might have been offered too late in the disease trajectory to be fully effective. This may have limited the potential for improvement in functional outcomes such as [I]ADL dependency, and may have reduced the relevance or applicability of certain psychoeducational components targeting behavioural changes and communication [53, 54]. The fact that a considerable number of persons with dementia was admitted to residential care or passed away during their participation in this study further reflects the advanced stage of the disease in this sample. However, a sensitivity analysis restricted to participants in milder dementia stages (GDS score 4 or lower) yielded broadly similar results, suggesting that disease stage alone may not fully explain the null findings, though the substantially reduced sample size in this subgroup limits the conclusions that can be drawn.

Finally, some of the dyads in this study were recruited through Meeting Centres, of which an unknown number might have participated in the Meeting Centres Support Programme, which offers structured social, creative, and physical activities for people with dementia alongside education and peer support for caregivers, and were thus already engaged in support services [25]. This might have contributed to the relatively high baseline scores observed in this sample and may have reduced the contrast between groups, thereby limiting the added effect of the MOMANT intervention. However, a sensitivity analysis restricted to the subsample of dyads recruited from settings other than Meeting Centres revealed comparable results, with trends in the same directions as the original analysis for the Pleasant Events Schedule and Positive Experience Scale. Additionally, earlier review studies found that many non-pharmacological interventions did not show significant effects on primary outcome measures [24, 45]. This raises important questions whether this reflects limited intervention effectiveness or rather the adequacy or sensitivity of commonly used outcome measures in dementia care research. The psychosocial processes targeted by the MOMANT intervention may not be fully captured by standard health-related quality of life instruments. Moreover, while between-group differences on standardised measures were limited, individual trajectories within the intervention group varied considerably, suggesting that some caregivers benefited more than others, with many caregivers reporting meaningful benefits. This notion is further supported by the generally positive subjective feedback from participants, who emphasised the value of practical guidance, peer recognition, and social support. Taken together, these findings suggest that objective outcome measures and subjective evaluations might capture different but complementary aspects of change, and both are important to understand the full impact of psychosocial interventions in clinical trials.

### Strengths and limitations

Several strengths of the MOMANT intervention and study design should be noted. First, the intervention materials were developed in consultation with caregivers, persons with dementia, and dementia care experts, ensuring that the content was both relevant and acceptable to the target population. Second, by collaborating with local healthcare and welfare organisations, the study could immediately be implemented in real world settings, allowing the manual and the intervention to remain easily in use after the study had ended. In total, the study worked together with 56 different organisations across the Netherlands, including those in rural regions that are typically underrepresented in (dementia) research. This broad inclusion contributed to the diversity and representativeness of the sample. Notably, a large proportion of the participants in the study had completed low to mid-level education, which is a characteristic of the current older generation and an important consideration when tailoring future interventions. Third, the intervention aimed to empower caregivers to take an active role in engaging the persons with dementia in meaningful activities, potentially strengthening caregiver autonomy beyond the intervention period. This empowerment was highlighted in the subjective evaluations through increased caregiver confidence. Such autonomy may be particularly valuable during situations where formal healthcare services are limited or disrupted, as was the case during the COVID-19 pandemic. Fourth, the intervention was person-centred, allowing caregivers to express their concerns and connect with peers, many of whom reported still being in contact 18–24 months after completion of the study, underscoring the importance of the lasting social impact of the group format.

Nonetheless, several limitations must be acknowledged. First, although the intervention was designed to be applicable for informal caregivers of community-dwelling persons with -dementia, many persons with dementia in the sample were already in a more advanced phase of the disease. Certain components of the intervention, like the psychoeducational components, may therefore have been offered too late to be optimally effective. Additionally, the average time since diagnosis was longer in the control group than in the intervention group, suggesting a possible imbalance in disease stage at baseline, which may partly explain the higher number of persons with dementia admitted to residential care in the control group during the study period. Moreover, a formal comparison of completers and non-completers on baseline characteristics was not conducted, which may limit the interpretation of attrition-related bias in the results. Second, although the sample was diverse in terms of region and educational background, participation in the intervention group might have been biased toward more motivated caregivers, potentially limiting the generalizability to more burdened or hard-to-reach populations. Although dyads in both conditions were recruited in the same way, the additional commitment required by the intervention condition may have made it more appealing to highly motivated caregivers. While efforts of recruitment were employed broadly, the number of participants from underrepresented groups, as for example ethnic minorities, was limited. Finally, the COVID-19 pandemic coincided with part of the follow up period of the study, possibly influencing outcomes such as caregiver well-being, perceived burden, as well as availability of services, potentially confounding intervention effects.

### Future research directions

Future research should further examine the mechanisms underlying the effects of psychosocial interventions and explore options to strengthen their impact. Although group-level effects of MOMANT compared to usual care were modest, individual trajectories within the intervention group varied, suggesting that some participants clearly benefited more than others. This underlines the importance of identifying for whom, when and under what conditions such interventions are most effective. Tailoring interventions to caregiver needs, relational dynamics, or stage of disease progression may enhance effectiveness. Another important direction for future research concerns the fact that standard outcome measures showed limited between-group differences, yet participants in the intervention group subjectively evaluated the MOMANT intervention as beneficial, enjoyable, and worth recommending with many participants maintaining social contact after the study. While this subjective feedback provides valuable insight into perceived relevance and acceptability, it does not necessarily reflect the same constructs as the objective outcome measures. The existing instruments may not fully capture all outcomes that caregivers themselves consider valuable [55], highlighting the need to critically reflect on and possibly expand the range of outcome measures in future research. This pattern aligns with other psychosocial intervention studies where subjective feedback is often positive despite limited objectively measurable change [56–58]. More sensitive or targeted outcome measures may better capture intervention effects, as standard tools often assess broad functions (e.g., quality of life or caregiver burden) that might be less likely to change [49, 59], and a mixed methods design may provide a richer impression of the intervention effects. Moreover, it is also important to consider the progressive and degenerative nature of dementia, as caregivers later in the disease trajectory may be too overburdened to participate in an intervention, further underlining the need for earlier diagnosis and support. Future research should also explore how timing and intensity of interventions influence both uptake and effectiveness across different stages of the disease.

### Clinical implications

The findings of this study have some practical implications. As a caregiver-led, low-threshold intervention, MOMANT would be suitable for implementation in community-based dementia care services, particularly in the early stages of the disease when proactive support is both critical and underutilized [60]. The manual-based format, the involvement of local healthcare and welfare professionals, and the person-centred nature of the sessions make it feasible to embed the intervention in routine care with minimal demand on healthcare professionals. Importantly, the positive experiences reported by caregivers regarding the opportunity for peer contact and personal support suggest that the intervention may also have value in promoting social connection, which may improve social health [61]. Despite an increasing recognition of the concept of social health as a crucial factor in the prevention and management of dementia, and its direct relevance to the daily life of the person with dementia and their informal caregiver, this concept remains underutilized in clinical practice [19, 61, 62]. MOMANT aligns well with this approach by promoting engagement in meaningful activities and emotional support in the home environment. However, the intervention may be more effective in the earlier stages of the disease trajectory, when proactive guidance and emotional support can help delay caregiver burden [19] and when stimulation of meaningful activities may help delay functional decline in the person with dementia [12].

## Conclusion

In conclusion, the MOMANT intervention did not result in significant improvements on most outcome measures. However, the intervention group did show a higher frequency of shared activities and more positive caregiving experience than the control group immediately after the intervention. Subjective evaluations were very positive, which raises the question whether future research should consider additional outcome measures that are more specific or sensitive to the outcomes targeted in the intervention. The study also showed that the MOMANT intervention demonstrated feasibility and acceptability as a caregiver-led intervention to stimulate the person with dementia in meaningful shared activities at home, as illustrated by the feedback from healthcare professionals and caregivers. Overall, this study underscores the demand among informal caregivers for information, emotional support, and services, and shows that MOMANT offers valuable insights for community-based dementia care, emphasizing a person-centred social approach.

## Supplementary Information

Supplementary Material 1.

Supplementary Material 2.

Supplementary Material 3.

Supplementary Material 4.

Supplementary Material 5.

Supplementary Material 6.

Supplementary Material 7.

## Data Availability

The datasets generated and/or analysed during the current study are available from the corresponding author on reasonable request.

## Abbreviations

AD: Alzheimer’s disease
ADL: Activities of daily living
CES-D1: Center for Epidemiologic Studies – Depression
CI: Confidence interval
COVID 19: Coronavirus disease 2019
DQOL: Dementia Quality of Life
EQ-5D-5L: EuroQol 5-Dimension 5-Level
GDS: Global Deterioration Scale
IADL: Instrumental activities of daily living
IDDD: Interview for Deterioration in Daily Living Activities in Dementia
MCI: Mild Cognitive Impairment
MOMANT: Mantelzorg Ondersteuning Met ActiviteiteN Thuis (Caregiver Support Programme with Activities at Home)
PES-AD: Pleasant Events Schedule-Alzheimer’s Disease
POS: POsitive experiences Scale
RUD: Resource Utilization in Dementia
SD: Standard deviation
SE: Standard error
SSCQ: Short Sense of Competence Questionnaire
VAS: Visual Analogue Scale
VU: Vrije Universiteit
ZONMW: ZorgOnderzoek Nederland Medische Wetenschappen (Dutch Organisation for Health Research and Care Innovation)
