# Supplementary material for: The MOMANT study, a caregiver support programme with activities at home for people with dementia: results of a randomised controlled trial
Source: BMC Geriatr. 2026 May 20;26:949. doi: 10.1186/s12877-026-07634-0 (PMC13366924; doi:10.1186/s12877-026-07634-0)
Supplement: Supplementary file 2 — Supplementary Material 2. [file 12877_2026_7634_MOESM2_ESM.docx]

Evaluation of the MOMANT intervention – 3 months

The first questions concern the handbook you received at the first session at the elderly care centre.

1. **Did you find the handbook useful?**

□ No, not useful at all □ Fairly useful

□ Somewhat useful □ Yes, very useful

1. **Did you learn anything new from the handbook?**

□ I have learned very little that was new □ I have learned several new things

□ I have learned a few new things □ I have learned many new things

1. **Were the information and the language used in the handbook easy to understand?**

□ No, it was not easy to understand □ Yes, most of it was easy to understand

□ No, most of it was not easy to understand □ Yes, everything was easy to understand

1. **Did you find the suggestions for activities in the handbook useful?**

□ No, not useful at all □ Fairly useful

□ Somewhat useful □ Yes, very useful

1. **How often do you think you used the advice and suggestions from the handbook at home?**

□ Less than once a month □ About once a week

□ About once a month □ More than once a week

1. **Did you miss any information in the handbook?**

□ No, the information was complete □ Yes, namely:

.………………………………………………………………………………….……………………………………..…………………………

The following questions are about engaging in activities at home. Whenever the questions mention “your relative”, this refers to your relative with dementia.

*If you have not engaged in any activities with your relative, you may go directly to question 13 (on the following page).*

1. **Approximately how much time do you think you have spent engaging in activities with your relative?**

□ Less than 1 hour per week □ More than 1 hour per week

□ About 1 hour per week □ At least 1 hour per day

1. **Do you think engaging in activities had a positive effect on your relative?**

□ No, no positive effect at all □ Fairly positive effect

□ Somewhat positive effect □ Yes, a very clear positive effect

1. **Do you think engaging in activities had a negative effect on your relative? (for example, it caused stress, irritation or confusion)**

□ No, no negative effect at all □ Fairly negative effect

□ Somewhat negative effect □ Yes, a very clear negative effect

1. **Do you think your relative enjoyed engaging in activities?**

□ No, not at all □ Often

□ Sometimes □ Yes, always

1. **Did you find it burdensome to engage in activities with your relative?**

□ No, not at all □ Often

□ Sometimes □ Yes, always

1. **Do you think engaging in activities with your relative was also beneficial for you?**

**(for example: it helped to structure your day, to keep your relative active, or to reduce your stress)**

□ No, it did not help me at all □ It helped me quite a bit

□ It helped me a little □ Yes, it helped me a lot

*If it helped you in any way, how did it help you?*

………………………………………………………………………………………………………………………………………………………………………………………………………………………………………………………………………………………………………………

1. **Did you have any difficulties engaging in activities at home? Or were there any reasons why engaging in activities was not possible?**

□ No, not at all □ Yes, namely:

………………………………………………………………………………………………………………………………………………………………………………………………………………………………………………………………………………………………………………

The following questions are about the sessions you attended at the local elderly care centre.

1. **Did you find the sessions useful?**

□ No, not useful at all □ Fairly useful

□ Somewhat useful □ Yes, very useful

1. **Did the healthcare professional who led the sessions explain the information clearly?**

□ No, it was not clear □ Yes, most of it was clear

□ No, most of it was not clear □ Yes, everything was clear

1. **Was the healthcare professional able to answer all questions clearly?**

□ No, most questions were not answered well

□ No, some questions were not answered well

□ Yes, most questions were answered well

□ Yes, all questions were answered well

□ No questions were asked

1. **Did you find it useful to share or exchange tips and experiences with other caregivers during the sessions?**

□ No, not useful at all □ Fairly useful

□ Somewhat useful □ Yes, very useful

The last two questions concern the intervention as a whole (this includes the sessions at the elderly care centre, the handbook, and the activities at home).

1. **Has the intervention changed the way you interact with your relative?**

□ No, not changed at all □ Changed quite a bit

□ Changed somewhat □ Yes, changed a lot

*If it has changed, in what way has it changed?*

………………………………………………………………………………………………………………………………………………………………………………………………………………………………………………………………………………………………………………

1. **Do you plan to continue using certain parts of the intervention or the activities at home?**

□ No, not at all □ Probably

□ Maybe □ Yes, definitely

*If yes, which parts do you plan to continue using at home?*

………………………………………………………………………………………………………………………………………………………………………………………………………………………………………………………………………………………………………………

**Do you have any further comments or suggestions?**

………………………………………………………………………………………………………………………………………………………………………………………………………………………………………………………………………………………………………………

Thank you very much for completing this questionnaire.
