# Supplementary material for: The MOMANT study, a caregiver support programme with activities at home for people with dementia: results of a randomised controlled trial
Source: BMC Geriatr. 2026 May 20;26:949. doi: 10.1186/s12877-026-07634-0 (PMC13366924; doi:10.1186/s12877-026-07634-0)
Supplement: Supplementary file 3 — Supplementary Material 3. [file 12877_2026_7634_MOESM3_ESM.docx]

Evaluation of the MOMANT intervention – 6 months

The first questions are about the handbook you received at the first session at the elderly care centre.

1. **Do you still use the handbook?**

□ No, not at all □ Yes, often

□ Yes, sometimes □ Yes, very often

*If you no longer use the handbook, you may skip question 2 and go directly to question 3.*

1. **Which parts of the handbook do you still use?**

□ The information section (Part A) □ The overview of websites and phone numbers of support services (Part C)

□ The activity section (Part B) □ I still use all parts (Part A, B, C)

The next question concerns the information and tips that you received during the intervention. These may include tips and advice discussed during the sessions or tips and advice you read in the handbook.

1. **Do you still use or apply any of the tips or advice that you received during the intervention?**

□ No, I no longer use the tips and advice □ Yes, I often use the tips and advice

□ Yes, I sometimes use the tips and advice □ Yes, I very often use the tips and advice

*If you still use or apply any of the tips or advice, could you briefly describe which ones you still use?*

…………………………………………………………………………………………………………………………………………………………………………………………………………………………………………………………………………………………………………………………

The following questions are about doing the activities at home. Whenever the question refers to “your relative”, this refers to your relative with dementia.

1. **Do you engage in activities with your relative?**

□ No, not at all □ Regularly

□ Sometimes □ Yes, quite often

1. **Have you started engaging in more activities with your relative as a result of the intervention?**

□ No, I engage in the same amount of activities □ Yes, I engage in slightly more activities

….as before the intervention than before the intervention

□ No, I even engage in fewer activities than □ Yes, I engage in a lot more activities than

before the intervention before the intervention

*If you do not engage in any activities with your relative at all, you may skip questions 6-10 and go directly to question 11.*

1. **Approximately how much time do you spend on engaging in activities with your relative?**

□ Less than 1 hour a week □ More than 1 hour a week

□ About 1 hour a week □ At least 1 hour a day

1. **When choosing or engaging in activities, do you use the suggestions from the handbook?**

□ No, not at all □ Often

□ Sometimes □ Yes, always

1. **Do you think your relative enjoys engaging in activities?**

□ No, not at all □ Often

□ Sometimes □ Yes, always

1. **Do you find it burdensome to engage in activities with your relative?**

□ No, not at all □ Often

□ Sometimes □ Yes, always

1. **Do you think that engaging in activities with your relative is also beneficial for you?**

**(for example, it helps to structure your day, keeps your relative active, or reduces your own stress)**

□ No, it does not help me at all □ Yes, it helps me quite a bit

□ Yes, it helps me a little □ Yes, it helps me a lot

*If it helps you in any way, could you maybe briefly explain your answer?*

………………………………………………………………………………………………………………………………………………………………………………………………………………………………………………………………………………………………………………

1. **Do you have any difficulties engaging in activities at home? Or are there any reasons why engaging in activities is not possible?**

□ No, not at all □ Yes, namely:

………………………………………………………………………………………………………………………………………………………………………………………………………………………………………………………………………………………………………………

The last question is about the intervention as a whole.

1. **Would you recommend this intervention to other caregivers?**

□ No, definitely not □ Yes, I think most caregivers could benefit from it

□ Only to caregivers who are heavily burdened □ Yes, I would definitely recommend this intervention to other caregivers

*Could you briefly explain your answer?*

………………………………………………………………………………………………………………………………………………………………………………………………………………………………………………………………………………………………………………

**Do you have any further comments or suggestions?**

………………………………………………………………………………………………………………………………………………………………………………………………………………………………………………………………………………………………………………

Thank you very much for completing this questionnaire!
