# Supplementary material for: The MOMANT study, a caregiver support programme with activities at home for people with dementia: results of a randomised controlled trial
Source: BMC Geriatr. 2026 May 20;26:949. doi: 10.1186/s12877-026-07634-0 (PMC13366924; doi:10.1186/s12877-026-07634-0)
Supplement: Supplementary file 4 — Supplementary Material 4. [file 12877_2026_7634_MOESM4_ESM.docx]

Evaluation of the training and guiding the sessions

– MOMANT study

The following questions are about the training that you received prior to the first session from one of the researchers (in most cases from Sanne Balvert).

1. **Do you feel that you received enough explanation and training before presenting the first session?**

□ No, did not receive enough explanation □ Somewhat enough explanation

□ Slightly too little explanation □ Yes, sufficient explanation

1. **Were the instructions and explanations in the training folder (received during the trainer’s visit) clear?**

□ No, not clear at all □ Mostly clear

□ Somewhat clear □ Yes, these were clear

The next questions are about the sessions themselves and how you experienced presenting them.

1. **Did you appreciate having some flexibility in how to shape the sessions yourself, or would you have preferred a more fixed programme?**

□ The flexibility was pleasant and I did not need a fixed programme

□ The flexibility was not pleasant, but a fixed programme would not have been helpful either

□ The flexibility was pleasant, but a little more structure would have been nice

□ The flexibility was not pleasant and I would have preferred a fixed programme

1. **During the sessions, were you able to discuss all the information from the handbook?**

□ No, very little from the handbook was discussed

□ No, only a small part of the handbook was discussed

□ Yes, most of the handbook was discussed

□ Yes, all topics from the handbook were discussed

1. **How confident did you feel in presenting the sessions?**

□ Not confident at all □ Sufficiently confident

□ Somewhat confident □ Completely confident

The following questions concern how you think the informal caregivers experienced the sessions.

1. **Do you think the informal caregivers benefited from the sessions?**

□ The caregivers did not benefit from the □ The caregivers benefited sufficiently
 sessions from the sessions

□ The caregivers benefited somewhat from the □ The caregivers benefited a lot

sessions from the sessions

**7. Do you think six sessions, presented over two months, were enough for the caregivers?**

□ No, there should be more than six □ Yes, six is exactly right

□ Six is fine, but there could also be more □ Six is too many, there could be fewer

*If you indicated fewer sessions, how many sessions do you think would be just right?*

……….. sessions

1. **Do you think there were certain topics missing that caregivers would have liked to discuss?**

□ No, all important topics were covered □ Yes, namely:

………………………………………………………………………………………………………………………………………………………………………………………………………………………………………………………………………………………………………………

1. **Do you think you were able to give enough personal attention during the sessions?**

□ No, not at all □ For the most part, yes

□ Somewhat □ Yes, there was enough personal attention

The following questions concern the handbook that the informal caregivers received during the first session.

1. **Do you think the caregivers found the information section (A) of the handbook useful?**

□ No, not useful □ Mostly useful

□ Somewhat useful □ Yes, very useful

1. **Do you think the caregivers found the activity section (suggestions and explanations for activities) useful?**□ No, not useful □ Mostly useful
   □ Somewhat useful □ Yes, very useful
2. **Do you think the step-by-step instructions for each activity were useful for the caregivers?**□ No, not useful □ Mostly useful
   □ Somewhat useful □ Yes, very useful
3. **Do you think the language used in the handbook was appropriate for the caregivers?**

□ No, not at all □ For the most part, yes

□ Somewhat □ Yes, it was appropriate

The following questions concern the home visit you made to each dyad. If you did not conduct any home visits, you may skip these questions and continue with question 17.

1. **Did you find the home visit a valuable addition to the intervention?**

□ No, not valuable at all □ Mostly valuable

□ Somewhat valuable □ Yes, very valuable

1. **Do you think the caregivers found the home visit a valuable addition?**

□ No, not valuable at all □ Mostly valuable

□ Somewhat valuable □ Yes, very valuable

1. **Do you think you were able to give enough personal attention during the home visit?**

□ No, not at all □ For the most part, yes

□ Somewhat □ Yes, there was enough personal attention

Lastly, the last two questions concern the intervention as a whole.

1. **Do you think the intervention is a useful addition to your field of work?**

□ Not useful □ Mostly useful

□ Somewhat useful □ Very useful

*Brief explanation:*

………………………………………………………………………………………………………………………………………………………………………………………………………………………………………………………………………………………………………………

1. **Do you think you will continue to use this intervention, or parts of it, in your field of work?**

□ No, I will not continue to use any part of it □ Yes, namely:

………………………………………………………………………………………………………………………………………………………………………………………………………………………………………………………………………………………………………………

**Do you have any further comments or suggestions?**

………………………………………………………………………………………………………………………………………………………………………………………………………………………………………………………………………………………………………………

Thank you so much for completing this questionnaire!
