# Supplementary material for: The MOMANT study, a caregiver support programme with activities at home for people with dementia: results of a randomised controlled trial
Source: BMC Geriatr. 2026 May 20;26:949. doi: 10.1186/s12877-026-07634-0 (PMC13366924; doi:10.1186/s12877-026-07634-0)
Supplement: Supplementary file 5 — Supplementary Material 5. [file 12877_2026_7634_MOESM5_ESM.docx]

| **Variable** | **Baseline score** | | **Time since diagnosis** | | **Age (PwD)** | | **AD** | | **MCI** | | **Centre type** | |  |
| --- | --- | --- | --- | --- | --- | --- | --- | --- | --- | --- | --- | --- | --- |
|  | **b (SE)** | ***p*** | **b (SE)** | ***p*** | **b (SE)** | ***p*** | **b (SE)** | ***p*** | **b (SE)** | ***p*** | **b (SE)** | ***p*** |  |
| **Outcome measure** | | | | | | | | | | | | |  |
| Quality of life (EQ-5D-5L) | 0.52 (0.04) | **<.01** | 0.00 (0.00) | .81 | -0.00 (0.00) | .67 | 0.02 (0.02) | .31 | 0.09 (0.05) | .053 | 0.01 (0.02) | .58 |  |
| Overall health (VAS) | 0.41 (0.06) | **<.01** | 0.00 (0.03) | .89 | 0.05 (0.12) | .67 | 0.17 (1.82) | .93 | -0.63 (4.15) | .88 | -0.98 (1.78) | .58 |  |
| CES-D total score | 0.11 (0.06) | .08 | 0.03 (0.03) | .44 | -0.07 (0.15) | .65 | 0.46 (2.25) | .84 | 5.89 (4.94) | .24 | -1.38 (2.16) | .53 |  |
| *Positive affect* | 0.71 (0.05) | **<.01** | 0.00 (0.00) | .28 | -0.00 (0.02) | .82 | -0.02 (0.23) | .92 | 0.16 (0.51) | .75 | -0.51 (0.22) | .**02** |  |
| *Depressed affect* | 0.71 (0.05) | **<.01** | 0.00 (0.00) | .55 | -0.03 (0.02) | .11 | 0.27 (0.31) | .39 | 0.95 (0.68) | .17 | -0.10 (0.30) | .75 |  |
| *Somatic symptoms* | 0.57 (0.06) | **<.01** | 0.01 (0.01) | .27 | -0.02 (0.03) | .64 | -0.39 (0.49) | .43 | 0.67 (1.09) | .54 | -0.61 (0.48) | .21 |  |
| *Interpersonal symptoms* | 0.53 (0.06) | **<.01** | 0.00 (0.00) | .88 | -0.00 (0.01) | .59 | 0.27 (0.12) | .**03** | 0.17 (0.26) | .52 | 0.16 (0.12) | .17 |  |
| SCQ total score | 0.65 (0.05) | **<.01** | 0.01 (0.01) | .27 | 0.04 (0.03) | .21 | 0.76 (0.53) | .16 | 0.82 (1.16) | .48 | 0.18 (0.51) | .73 |  |
| *Consequences for personal life* | 0.59 (0.06) | **<.01** | 0.00 (0.00) | .93 | -0.00 (0.02) | .74 | -0.29 (0.29) | .31 | -0.10 (0.63) | .87 | -0.12 (0.28) | .66 |  |
| *Satisfaction as caregiver* | 0.58 (0.05) | **<.01** | 0.01 (0.00) | .10 | 0.03 (0.02) | .052 | 0.80 (0.26) | **.002** | 0.47 (0.58) | .42 | 0.23 (0.25) | .37 |  |
| *Satisfaction with PwD* | 0.58 (0.07) | **<.01** | 0.00 (0.00) | .82 | 0.02 (0.01) | .09 | 0.37 (0.20) | .07 | 0.47 (0.45) | .30 | 0.11 (0.20) | .58 |  |
| Positive Experiences | 0.50 (0.06) | **<.01** | 0.00 (0.00) | .40 | 0.01 (0.01) | .50 | -0.25 (0.19) | .18 | -0.77 (0.41) | .06 | -0.16 (0.18) | .38 |  |
| DQOL *Positive affect* | 0.48 (0.07) | **<.01** | 0.01 (0.01) | .60 | 0.04 (0.04) | .29 | 0.03 (0.58) | .96 | -3.14 (1.17) | .**01** | 0.08 (0.57) | .90 |  |
| DQOL *Negative affect* | 0.71 (0.09) | **<.01** | -0.00 (0.02) | .89 | 0.03 (0.07) | .73 | 0.44 (1.08) | .68 | -3.05 (2.47) | .22 | -0.47 (1.05) | .65 |  |
| DQOL *Sense of belonging* | 0.41 (0.07) | **<.01** | 0.00 (0.01) | .96 | -0.02 (0.02) | .47 | -0.03 (0.32) | .91 | 0.71 (0.67) | .29 | -0.19 (0.31) | .55 |  |
| DQOL *Aesthetics* | 0.71 (0.07) | **<.01** | 0.00 (0.01) | .88 | -0.04 (0.04) | .30 | 0.29 (0.61) | .64 | -2.54 (1.43) | .08 | 0.76 (0.59) | .20 |  |
| DQOL *Self esteem* | 0.37 (0.08) | **<.01** | -0.00 (0.01) | .59 | -0.01 (0.03) | .72 | 0.53 (0.43) | .23 | 1.38 (1.01) | .18 | 0.32 (0.43) | .46 |  |
| PES Enjoyment | 0.64 (0.07) | **<.01** | -0.00 (0.01) | .72 | -0.05 (0.05) | .30 | 1.05 (0.80) | .18 | -0.73 (1.62) | .65 | 1.22 (0.73) | .10 |  |
| PES Frequency | 0.59 (0.07) | **<.01** | -0.02 (0.01) | .13 | -0.09 (0.05) | .09 | 0.59 (0.84) | .48 | 0.29 (1.76) | .87 | 0.22 (0.78) | .77 |  |
| IDDD ADL | 0.73 (0.06) | **<.01** | -0.00 (0.02) | .94 | 0.03 (0.07) | .67 | 0.19 (1.04) | .86 | 1.68 (2.31) | .47 | -0.64 (1.01) | .53 |  |
| IDDD [I]ADL | 0.30 (0.08) | **<.01** | 0.01 (0.02) | .51 | -0.09 (0.08) | .24 | -0.92 (1.23) | .45 | -0.15 (2.73) | .96 | -0.44 (1.19) | .71 |  |

**Supplementary Table 1: Fixed estimates from the covariates, based on the results from the longitudinal mixed models**

Note. SE = standard error, PwD = Person with Dementia, AD = Alzheimer’s Disease, MCI = Mild Cognitive Impairment, VAS = visual analogue scale, CES-D = Center for Epidemiologic Studies Depression scale, SCQ = Sense of Competence, DQOL = Dementia Quality Of Life, PES= Pleasant Events Schedule, IDDD = Interview for Deterioration in Daily Living Activities in Dementia
