# Supplementary material for: The MOMANT study, a caregiver support programme with activities at home for people with dementia: results of a randomised controlled trial
Source: BMC Geriatr. 2026 May 20;26:949. doi: 10.1186/s12877-026-07634-0 (PMC13366924; doi:10.1186/s12877-026-07634-0)
Supplement: Supplementary file 6 — Supplementary Material 6. [file 12877_2026_7634_MOESM6_ESM.docx]

| **Quality of life (EQ-5D-5L)** | **Baseline** | | **3 months** | | **6 months** | |
| --- | --- | --- | --- | --- | --- | --- |
|  | **Intervention** | **Control** | **Intervention** | **Control** | **Intervention** | **Control** |
| Mobility |  |  |  |  |  |  |
| 1 | 68.9% | 72.4% | 70.8% | 71.6% | 62.1% | 68.3% |
| 2 | 12.2% | 4.1% | 10.8% | 12.2% | 19.0% | 14.3% |
| 3 | 14.9% | 14.3% | 13.8% | 9.5% | 15.5% | 9.5% |
| 4 | 4.1% | 9.2% | 4.6% | 6.8% | 3.4% | 7.9% |
| 5 | - | - | - | - | - | - |
| Self-care |  |  |  |  |  |  |
| 1 | 94.6% | 94.9% | 96.9% | 97.3% | 100.0% | 98.4% |
| 2 | 4.1% | 1.0% | 1.5% | 2.7% | - | 1.6% |
| 3 | 1.4% | 1.0% | 1.5% | - | - | - |
| 4 | - | 2.0% | - | - | - | - |
| 5 | - | 1.0% | - | - | - | - |
| Usual activities |  |  |  |  |  |  |
| 1 | 85.1% | 72.4% | 92.3% | 73.0% | 82.8% | 68.3% |
| 2 | 8.1% | 11.2% | 3.1% | 10.8% | 8.6% | 14.3% |
| 3 | 4.1% | 9.2% | 3.1% | 10.8% | 6.9% | 12.7% |
| 4 | 2.7% | 6.1% | 1.5% | 5.4% | 1.7% | 4.8% |
| 5 | - | 1.0% | - | - | - | - |
| Pain/discomfort |  |  |  |  |  |  |
| 1 | 54.1% | 53.1% | 53.8% | 56.8% | 48.3% | 49.2% |
| 2 | 24.3% | 17.3% | 24.6% | 10.8% | 22.4% | 17.5% |
| 3 | 14.9% | 21.4% | 21.5% | 27.0% | 24.1% | 28.6% |
| 4 | 6.8% | 7.1% | - | 5.4% | 5.2% | 3.2% |
| 5 | - | 1.0% | - | - | - | 1.6% |
| Anxiety/depression |  |  |  |  |  |  |
| 1 | 78.4% | 78.6% | 80.0% | 81.1% | 82.8% | 74.6% |
| 2 | 17.6% | 14.3% | 15.4% | 8.1% | 10.3% | 12.7% |
| 3 | 4.1% | 2.0% | 3.1% | 9.5% | 6.9% | 11.1% |
| 4 | - | 4.1% | 1.5% | 1.4% | - | 1.6% |
| 5 | - | 1.0% | - | - | - | - |

**Supplementary Table 2: Individual dimension scores of the EQ-5D-5L over time**

Note. Data depicted in percentages. Scores range from 1 (no problems) to 5 (extreme problems).
