# Supplementary material for: The MOMANT study, a caregiver support programme with activities at home for people with dementia: results of a randomised controlled trial
Source: BMC Geriatr. 2026 May 20;26:949. doi: 10.1186/s12877-026-07634-0 (PMC13366924; doi:10.1186/s12877-026-07634-0)
Supplement: Supplementary file 7 — Supplementary Material 7. [file 12877_2026_7634_MOESM7_ESM.docx]

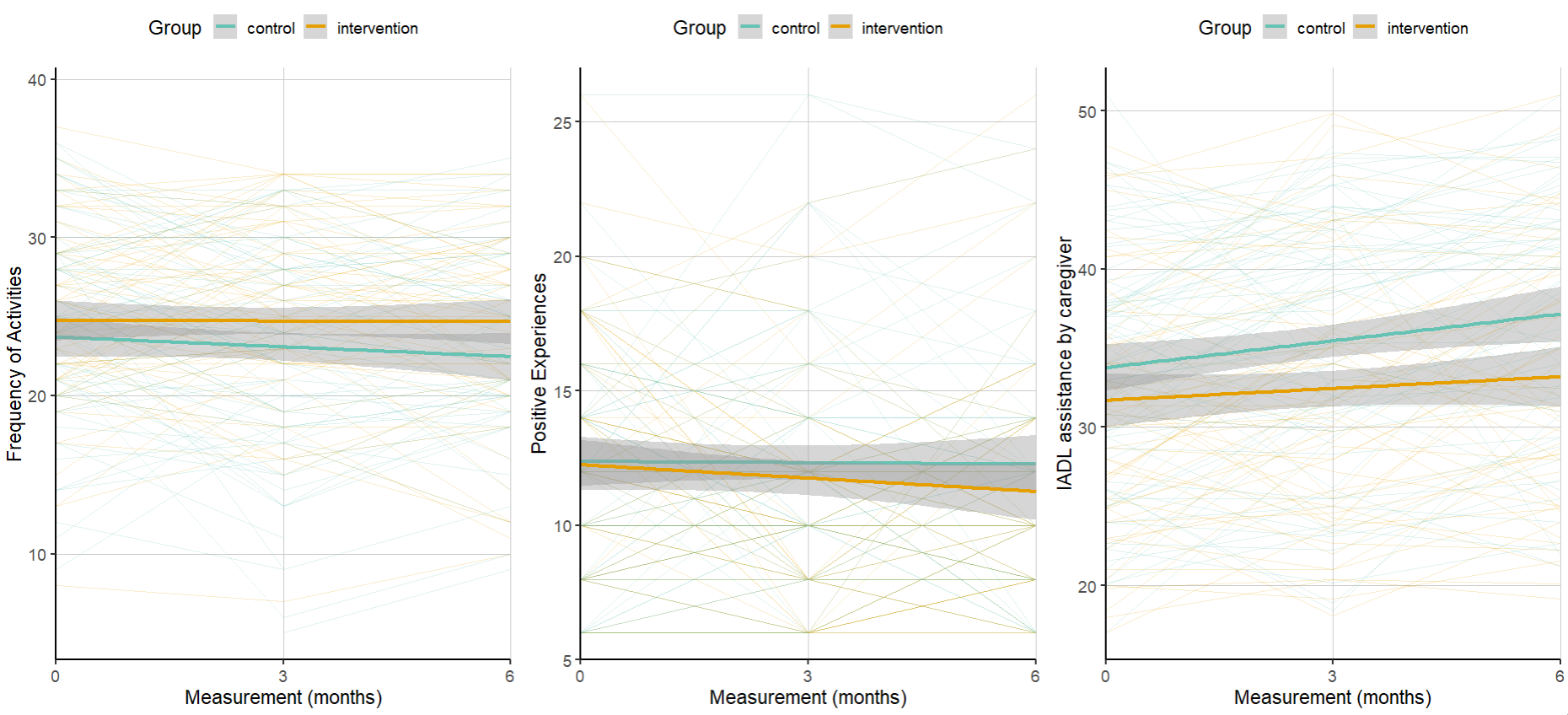

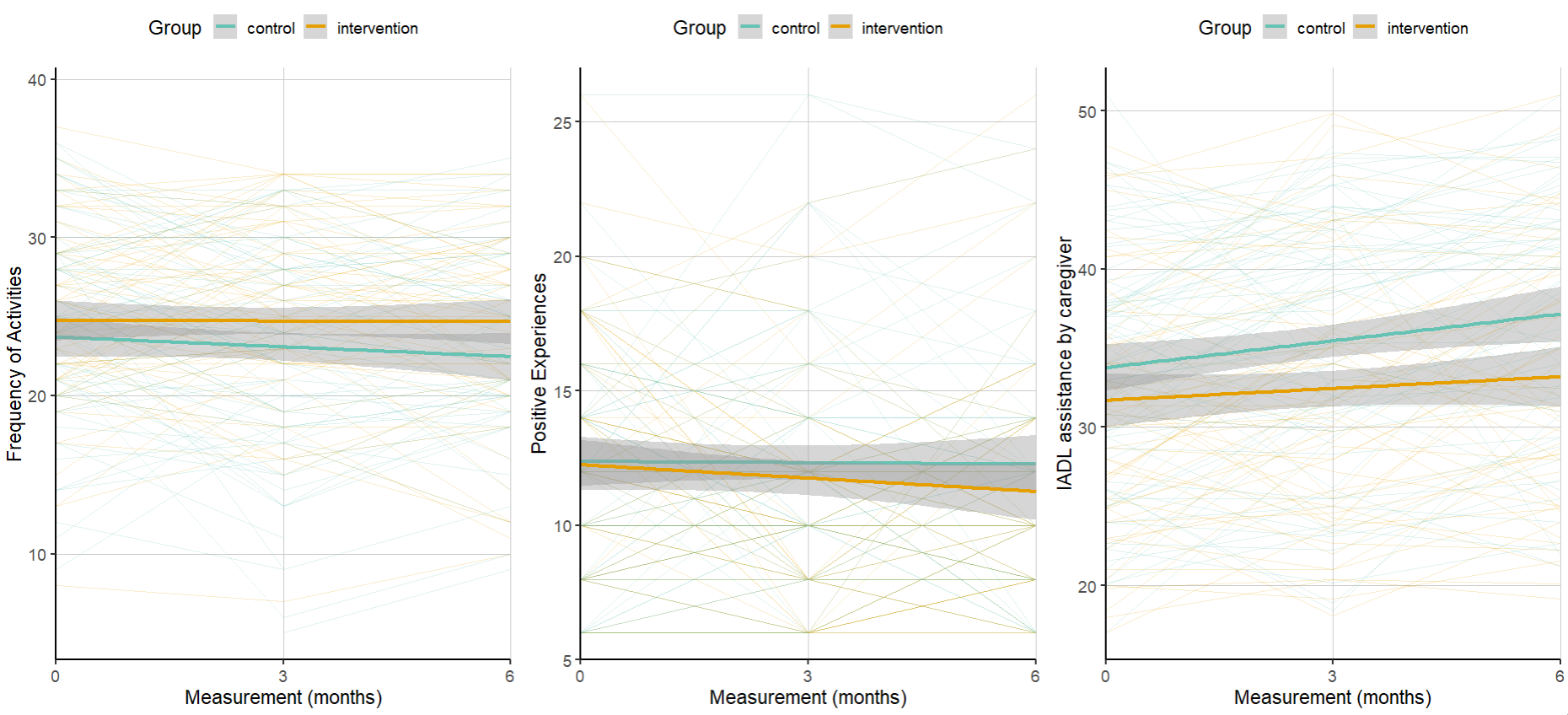


**Supplementary Figure 1. Scores on the frequency of activities scale and positive experiences scale over time.**

*Note: The thin lines indicate the trajectories of the scores of individual participants, the thick lines indicate the mean trajectory of the control and intervention group, with 95% CI in grey. This figure does not display the intervention effects as analysed in the manuscript, but only raw scores over time.*
